# Molecular characterization of zinc metalloproteinase Nas-14 from *Trichinella spiralis* and its participation in intestinal invasion

**DOI:** 10.1371/journal.pntd.0013437

**Published:** 2025-08-21

**Authors:** Qingbo Lv, Guangquan Si, Chengyao Li, Ning Jiang, Yushu He, Zijian Dong, Hanhai Mao, Mingyuan Liu, Xiaolei Liu, Ying Zhao, Jing Ding

**Affiliations:** 1 State Key Laboratory for Diagnosis and Treatment of Severe Zoonotic Infectious Diseases, Key Laboratory for Zoonosis Research of the Ministry of Education, Institute of Zoonosis, and College of Veterinary Medicine, Jilin University, Changchun, China; 2 China Conservation and Research Center for the Giant Panda, Key Laboratory of SFGA on the Giant Panda, Chengdu, Sichuan, China; 3 Jiangsu Co-innovation Center for Prevention and Control of Important Animal Infectious Diseases and Zoonoses, Yangzhou, Jiangsu, China; 4 Department of Nephrology, The First Hospital of Jilin University, Changchun, China; University of Passo Fundo: Universidade de Passo Fundo, BRAZIL

## Abstract

Astacins, a family of zinc metalloproteinases, are involved in invasion and tissue migration processes in a variety of parasites. An astacin-like proteinases have been detected in the excretory-secretory products (ESPs) of *Trichinella spiralis* (*T. spiralis*), zinc metalloproteinase Nas-14 (TsNas14), but its function in *T. spiralis* remains unclear. The primary objective of this research was to delineate the molecular characterization of TsNas14 and explore its potential to compromise the integrity of the intestinal barrier. Results showed that TsNas14 contains an Astacin domain and two ShK domains. It is highly conserved and has a consistent transcriptional expression pattern in the *Trichinella* genus. Quantitative results showed that the TsNas14 is transcribed and expressed during the whole life cycle, but that the expression level was highest in the adult worm (AW) stage. In the 3d AW stage, TsNas14 is mainly distributed on the stichosome, ovary, cuticle, and hypodermis, while in the 6d AW stage, it is only present on the cuticle. Gelatin zymography showed that the oligomerized rTsNas14 had the enzyme activity to degrade gelatin, and could be effectively inhibited by 1,10-Phenanthroline, indicating that it had the natural activity of metalloproteinases. In vitro experiments showed that rTsNas14 can down-regulate the expression of occludin and claudin-1 proteins of human colorectal adenocarcinoma (Caco-2) cells and improve the permeability of an intestinal barrier model. In addition, the direct incubation of rTsNas14 with claduin-1 showed that rTsNas14 could significantly degrade claudin-1. In vivo studies have demonstrated that inhibition of TsNas14 expression significantly impairs the infectivity of *T. spiralis* in mice, resulting in a decreased AW and muscle larvae burden. These findings suggest that TsNas14 plays a crucial role in *T. spiralis* intestinal invasion and may serve as a novel potential target for *Trichinella* vaccines or therapeutic interventions.

## Author summary

We report the functional characterization of TsNas14, a zinc metalloprotease critical for *Trichinella spiralis* (*T. spiralis*) invasion. This secretory protein features an Astacin catalytic domain flanked by two ShK toxin-like domains, with high evolutionary conservation across *Trichinella* species. TsNas14 was highly expressed specifically during the adult stage, and this expression pattern was highly consistent with the intestinal invasion stage of the parasite. To clarify the biological function of TsNas14 during the host invasion process, we successfully achieved the recombinant expression of this protein and verified its enzymatic activity. Recombinant TsNas14 demonstrated potent proteolytic activity against intestinal tight junction proteins and disrupted intestinal barrier models, mechanistically explaining its role in breaching host defenses. Crucially, RNAi silencing of TsNas14 reduced larvae and adult worm burdens in vivo. In conclusion, this study has for the first time revealed the key role of TsNas14 in the pathogenic process of *T. spiralis*. This discovery not only expands the understanding of the mechanism of parasitic invasion but also provides a new molecular target for the vaccine development and targeted therapy of *T. spiralis*.

## 1. Introduction

*Trichinella spiralis* (*T. spiralis*) is a global food-borne zoonotic parasite [1]. In China, the consumption of raw pork is the most common cause of trichinellosis in Yunnan, Guangxi, and Tibet, leading to serious public health threats and economic issues in food production [2,3]. Molecules secreted by *T. spiralis* during infestations have long been considered natural targets for promising drugs or vaccine development and understanding the molecular mechanism of *T. spiralis* invasion is of great significance for preventing animal infection and protecting public food safety.

*T. spiralis* develops through four stages in the same host but in different ecological niches. Briefly, the host ingests raw meat infected with muscle larvae (ML), which are released under the action of digestive juices and then burrow into the small intestine to begin development into intestinal infectious larvae (IIL). These undergo four molts to develop into adult worms (AW), a process that takes only about 30 hours [4]. The adult female then mates with an adult male to produce newborn larvae (NBL) about five days after infection. The NBL burrows into the intestinal wall and travels throughout the body with the blood or lymphatic vessels, eventually settling in skeletal muscle, to develop into ML [4]. At this stage, the host is considered chronically infected [4]. During the initial phase of infection, the AW must penetrate the intestinal lamina propria to establish successfully, which is critical for their survival. Appropriately, the excretory-secretory products (ESPs) of *T. spiralis* contain protein molecules conducive to tissue migration and invasion, such as cathepsin B, serine protease, and metalloproteinases [5–7]. With the development of genome sequencing and mass spectrometry techniques, the complex components in ESPs can be characterized with high throughput [8,9] and can potentially serve as targets for the development of new drugs and vaccines.

Nematode astacins (Nas) is a family of zinc metalloproteinases conserved in nematodes and belonging to the peptidase family M12 [10]. Members of this family all contain an astacin domain with zinc binding (HEXXHXXGFXHEXXRXDR) and Met-turn (SXMHY) motifs [11]. It is one of the representative protein families in nematode secretions, involved in various biological processes related to parasitism including parasite invasion, molting, development, reproduction, and immune escape [5,12–16]. *Strongyloides stercoralis* secretes an astacin-like protein that helps worms infect and migrate to the host’s gut [12], while *Onchocerca volvulus* relies on a stroma-degrading astacin-like protein to migrate within the host tissue [17]. Also, inhibition of Nas-36 and Nas-37 results in molting defects and a temperature-sensitive lethal phenotype in *Caenorhabditis elegans* [18], while nematodes lacking Nas-35 have a dumpy appearance [14]. Chemical inhibition of Nas-35 in *Brugia malay* and *Teladorsagia circumcincta* caused a severe dumpy phenotype [14,19], while inhibition of Nas-33 can induce molting defects and death in *Haemonchus contortus* infective larvae [13]. Thus, Nas can serve as drug targets for parasitic nematodes of socioeconomic importance.

In a previous transcriptome and proteome study, we identified a secreted protein present in the ESPs of *T. spiralis*, named *T. spiralis* zinc metalloproteinase Nas-14 (TsNas14), a member of the Nas family that is highly expressed during the intestinal invasion phase of the AW [8,20]. This protein is highly conserved within the *Trichinella* genus and exhibits a similar pattern of transcriptional expression level across different species, suggesting that TsNas14 is a crucial protease in its biological processes. To explore the role of this protein in the intestinal invasion process of *T. spiralis*, this study constructed a recombinant TsNas14 protein (rTsNas14) with enzyme activity, characterized its biological and biochemical characteristics, and initially explored its degradation of tight junction protein in an intestinal epithelial barrier model. Furthermore, we employed RNA interference (RNAi) technology and conducted in vivo experiments to examine the impact of TsNas14 on the infectivity of *T. spiralis*. The characterization of TsNas14 highlights this conserved *Trichinella*-specific gene family as a promising candidate target for the development of novel vaccines and therapeutic agents.

## 2. Materials and methods

### 2.1. Ethics statement

All animal experiments were approved by the Ethics the Ethical Committee of Jilin University affiliated with the Provincial Animal Health Committee (Changchun, China) (ethical clearance KT202202140).

### 2.2. Parasites and animals

The *T. spiralis* used in this study was from the ISS534 strain preserved in the laboratory and maintained in ICR mice. The ML, IIL, and AW were collected as previously reported [20]. Then, 6d AW was cultured in RPMI-1640 medium with 5% CO_2_ at 37°C for 16 h, removed with a 100 μm filter, and the NBL collected by centrifugation. The experimental hosts were ICR female mice aged 6–8 weeks and New Zealand rabbits, both purchased from Yisi Laboratory Animal Technology Co., LTD (Changchun, China).

### 2.3. Sequence analysis

ProtParam (https://web.expasy.org/protparam/) [21] was used to calculate various physical and chemical parameters of the amino acid sequence of TsNas14, while DeepTMHMM (https://dtu.biolib.com/DeepTMHMM/) [22] was used to predict the signal peptide and transmembrane structure of the protein. The domain information of the protein was obtained by InterPro web service (https://www.ebi.ac.uk/interpro/) [23] and SMART (https://smart.embl.de/) [24] prediction. The 3D conformation of the TsNas14 protein was predicted using the AlphaFold3 (https://golgi.sandbox.google.com/) [25] online service and the pTM scores were obtained. The quality of the predicted 3D conformations was evaluated using the SAVE v6.1 (https://saves.mbi.ucla.edu/) server [26–28] and amino acid phylogenetic tree of TsNas14 and homologous proteins from other species was constructed using RAxML [29]. To investigate the transcriptional signature of the TsNas14 homologs in other *Trichinella* sister species, we reanalyzed already published transcriptome data (GSE140382) [20]. Finally, bowtie2 [30] was used to map the short-reads sequence to the CDS region of TsNas14 homologue genes to calculate relative abundance.

### 2.4. Preparation of ESPs

The ML, AW, and NBL were cultured in RPMI-1640 medium (Gibco) containing 2% penicillin-streptomycin for 18 h, and the culture medium was collected and filtered with a 0.22 μm filter. The clarified culture medium was concentrated using the 3 kDa ultrafiltration centrifuge tube from Sangon Biotech (Shanghai, China) to obtain the ESPs protein of *T. spiralis*, and phosphate buffer solution (PBS) was used to replace the medium components.

### 2.5. Acquisition of rTsNas14 and anti-rTsNas14 serum

The TsNas14 gene (GenBank: KAL1234369.1) without a signal peptide was cloned, and the recombinant expression plasmid pET-28a/TsNas14 was transferred to *E. coli* BL21 (DE3). The protocol of prokaryotic expression was based on previous studies [31]. The expression was induced by 1 mM isopropyl β-D-1-thiogalactoside (IPTG) at 37°C for 6 h and the induced expression of *E. coli* was broken by ultrasonic crusher (SCIENTZ, China). After centrifugation, the crushed supernatant and precipitation were sampled separately for an SDS-PAGE test. The inclusion bodies were dissolved with 8 M urea and purified by Ni-NTA Sepharose 6FF (His-Tag) by Sangon Biotech (Shanghai, China). The purified product was placed in a semi-permeable membrane to slowly reduce the urea concentration to fold protein and to restore its activity until the urea concentration in the inclusion body was below 2 M and the soluble protein was considered successful in its renaturation. A Chromogenic LAL Endotoxin Assay Kit (Beyotime Biotechnology, China) was used to detect the endotoxin content in the protein products and the resultant recombinant protein was termed rTsNas14.

Based on previous studies, a New Zealand rabbit was immunized with 500 μg rTsNas14 emulsified with complete Freund’s adjuvant at a subcutaneous multisite [31]. Two weeks later, rTsNas14 protein emulsified with an equal amount of incomplete Freund’s adjuvant was administered twice every two weeks. After the last immunization, rabbit auricular venous blood was collected and the anti-rTsNas14 serum was isolated. Blood from rabbits vaccinated only with Freund’s adjuvant was also collected as a negative control and the anti-rTsNas14 IgG titers were determined by indirect ELISA using rTsNas14 as the coated antigen.

### 2.6. Enzymatic determination

The activity of rTsNas14 was determined by gelatin zymography. First, rTsNas14 was added with non-denatured loading buffer (Beyotime Biotechnology, China) and pig gelatin as substrate, and electrophoresis was performed in 10% non-denatured acrylamide gel. Blank (loading buffer only) and inhibitor controls (rTsNas14 and 1,10-phenanthroline) were also set up for electrophoresis. After the gel was cut and placed in the incubation solution for 48 h, it was stained with Coomassie brilliant blue dye for 30 min and decolorized with decolorizing solution for 2 h to observe whether the gel had transparent bands indicative of enzyme activity.

### 2.7. Cell culture and treatment

The human colorectal adenocarcinoma (Caco-2) cells were cultured in Dulbecco’s modified eagle medium (DMEM, Gibco), 10% Fetal Bovine Serum (FBS, Gibco), 1% penicillin-streptomycin (Solarbio, Beijing, China) and 5% CO_2_ for 48 h. After three stable passages, cells were utilized for subsequent experiments when reaching 70–80% confluence in culture dishes. Before starting the experiment, the cell medium was replaced with serum-free medium to prevent possible interference caused by FBS. The experimental design comprised four treatment groups: 1) Control group received inactive protein (1 μg/mL) of *T. spiralis* purified from the same expression vector as rTsNas14; 2) Positive control group was treated with LPS (20 μg/mL); 3) Recombinant protein group received rTsNas14 (1 μg/mL); 4) Inhibitor group was administered with both 1 μg/mL rTsNas14 and 10 μM 1,10-phenanthroline. All groups underwent continuous incubation for 12 hours post-intervention. Then, RIPA lysate (Solarbio, Beijing, China) was added to lyse the cells on ice for 30 min, and centrifuged at 12,000 × g for 10 min to collect supernatants.

### 2.8. Detection of rTsNas14 effects on occludin and claudin-1

Caco-2 cells were lysed using RIPA buffer and centrifuged at 12,000 × g for 10 min to collect supernatants. Protein concentrations were quantified with a BCA assay kit (UUBio, Suzhou, China). Aliquots containing 1 mg/mL total protein were co-incubated with either control protein or 1 μg/mL rTsNas14 at 37°C for 6 h. Protein levels of occludin and claudin-1 were analyzed by western blot.

### 2.9. Cytotoxicity detection of rTsNas14

The effect of rTsNas14 on the viability of Caco-2 cells was detected with a cell counting kit-8 (CCK-8) assay (Apexbio, USA). The Caco-2 cells were inoculated in 96-well cell culture plates at 5000/well for 24 h. Then, 10 μL of rTsNas14 at different concentrations was added until the final concentration was 0, 0.5, 1, 2, and 4 μg/mL, respectively, and incubated at 37°C for 24 h and 48 h. Thereafter 10 μL of CCK-8 detection solution was added to each well and after incubation at 37°C for 1 h, the absorbance was measured at 450 nm by a microplate reader (Biotek, USA).

### 2.10. Establishment of intestinal epithelial barrier model and integrity detection

The Caco-2 cells were inoculated into a 12-well Transwell culture plate (Biofil, Guangzhou China) with a density of 1 × 10^5^ cells/well, cultured in a 5% CO_2_ incubator at 37°C, and grown to full confluency. Transepithelial electrical resistance was used to assess the integrity of the monolayer and the change in trans-epithelial electrical resistance (TEER) was monitored by Millicell-ERS2 (Millipore USA) every two days [6]. When the epithelial resistance value was stable at 1000 Ω•cm^2^, the cell barrier was treated with rTsNas14 (1 μg/ml), 1,10-Phenanthroline pretreated rTsNas14 (1 μg/mL) and lipopolysaccharide (LPS) for 48h. The LPS alone was used as a positive control, while the cell medium was used as a negative control. There were three independent replicates in each group and a cell-free blank well in each experiment and the TEER value was calculated as follows: TEER = (Ω_measured_ - Ω_blank_) × film bottom area. Each well was measured three times at different positions and the average value was taken.

### 2.11. Quantitative real-time PCR

The total RNA of the seven different developmental stages of *T. spiralis* (ML, IIL 6 h, IIL 12 h, IIL 30 h, AW 3 d, AW 6 d, and NBL) was extracted with Trizol reagent (Takara, Japan). The specific primers (TsNas14-F: 5’-ATGTGCTGATCAAGATTGGGGT-3’; TsNas14-R: 5’-GACCCTGTTTGACCCACCAA-3’) were synthesized by Sangon Biotech (Shanghai, China) and qPCR was performed. The tubulin beta chain (GenBank ID: XM_003369432) gene of *T. spiralis* was sequenced as an internal parameter with the β-Tubulin-F:5’-GCTATTTGACAGTGGCTGCG-3’ and β-Tubulin-R: 5’- TTGAATGGCGGTCGAATTGC-3’ primers. The expression of TsNas14 was evaluated by 2^−ΔΔCt^.

### 2.12. Western blot analysis

The level of rTsNas14-induced expression in *E. coli* was detected using the rabbit His-Tag monoclonal antibody (Servicbio, China) as a primary antibody and the goat anti-rabbit IgG as a secondary antibody. The expression levels of occludin and claudin-1 were detected by using mouse claudin-1, occludin monoclonal antibody (Affinty, USA) as primary antibody, and goat anti-mouse IgG antibody as the secondary antibody. The mouse anti-β-actin monoclonal antibody was selected as the internal reference protein and the expression levels of TsNas14 in ESPs and worms were detected using a self-made rabbit anti-rTsNas14 polyclonal antibody serum as the primary antibody and goat anti-rabbit IgG antibody as the secondary antibody. The concentrations of all protein samples were quantified before analysis.

### 2.13. Immunofluorescence detection

The immunofluorescence detection of *T. spiralis* was improved upon the basis of previous studies [32]. The AW, ML, and NBL of *T. spiralis* were collected and fixed in 4% paraformaldehyde for 48 h where after precooled methanol was added at 4°C to fix the worms for 5 min. Then 1% TritonX-100 was added and the samples were incubated for 8 h at room temperature (RT) and 5% goat serum was used to block the worm body at RT for 1 h. The anti-rTsNas14 serum was added at 1:200 and samples were incubated at 4°C for 12 h. Alexa Fluor 555 secondary antibody (Abcam, Britain) was added at 1:1000 and the samples were incubated at RT for 40 min. Then DAPI Fluoromount-G (Yeasen, Shanghai China) was added and the samples were incubated at 4°C away from light for 12 h. Each step was followed with three PBS washes. The results were observed under a fluorescence microscope and a laser confocal microscope.

### 2.14. siRNA-mediated RNA interference

Based on the RNAi interference method used in previous studies [33], the TsNas14 gene was knocked down. TsNas14 specific siRNA, TsNas14-siRNA-721 (5’ → 3’ UAAAUUGUCGUAGCAUUGCT), TsNas14-siRNA-465 (5’ → 3’ UUGUUGAUAUCGAUGUAGCT) and TsNas14-siRNA-115 (5’ → 3’ UGCAAAUAGUCGUAGAGCT) was designed based on the full-length TsNas14 cDNA sequence and synthesized by Sangon Biotechnology (Shanghai, China). An unrelated siRNA sequence (NC-cy3: 5’ → 3’ ACGUGACACGUUCGGAGAATT) labeled with cy3 fluorescence was also synthesized as a negative control. After incubation with Lipo2000 (Thermo Fisher, USA), the siRNA was transfected into MLs by electroporation using a Gene Pulser Xcell System (Bio-Rad, USA) and incubated in RPM1640 culture medium for 36 h at 37°C with 5% CO_2_. As previously mentioned, the transcription and protein expression levels of TsNas14 in siRNA-treated and control were analyzed by qPCR and Western blot, respectively.

The MLs treated with siRNA were used to artificially infect mice to evaluate the infective capacity and fecundity of the transfected larvae. Forty-five ICR mice were randomly divided into three groups. In brief, the Blank group (n = 15) was oral gavage with 300 MLs untreated, the NC group (n = 15) was oral gavage with 300 MLs transfected with NC siRNA, and the 721 group (n = 15) was oral gavage with 300 MLs transfected with Ts-nas14–721 siRNA. Five mice were sacrificed at 3-, 6-, and 35 days post-infection (dpi) respectively to calculate the AWs and MLs infection loads (excluding the mice that died during the experiment), in order to assess the infectivity. Fecundity was assessed by the 24-hour NBL production of a single female worm.

### 2.15. Statistical analysis

All data were analyzed using GraphPad Prism 8.0 and the values were expressed as mean ± standard deviation (SD). One-way ANOVA was used to compare the differences between the groups and *P* < 0.05 was considered statistically significant.

## 3. Results

### 3.1. TsNas14 is a conserved protein in *Trichinella* species

**Fig 1:**
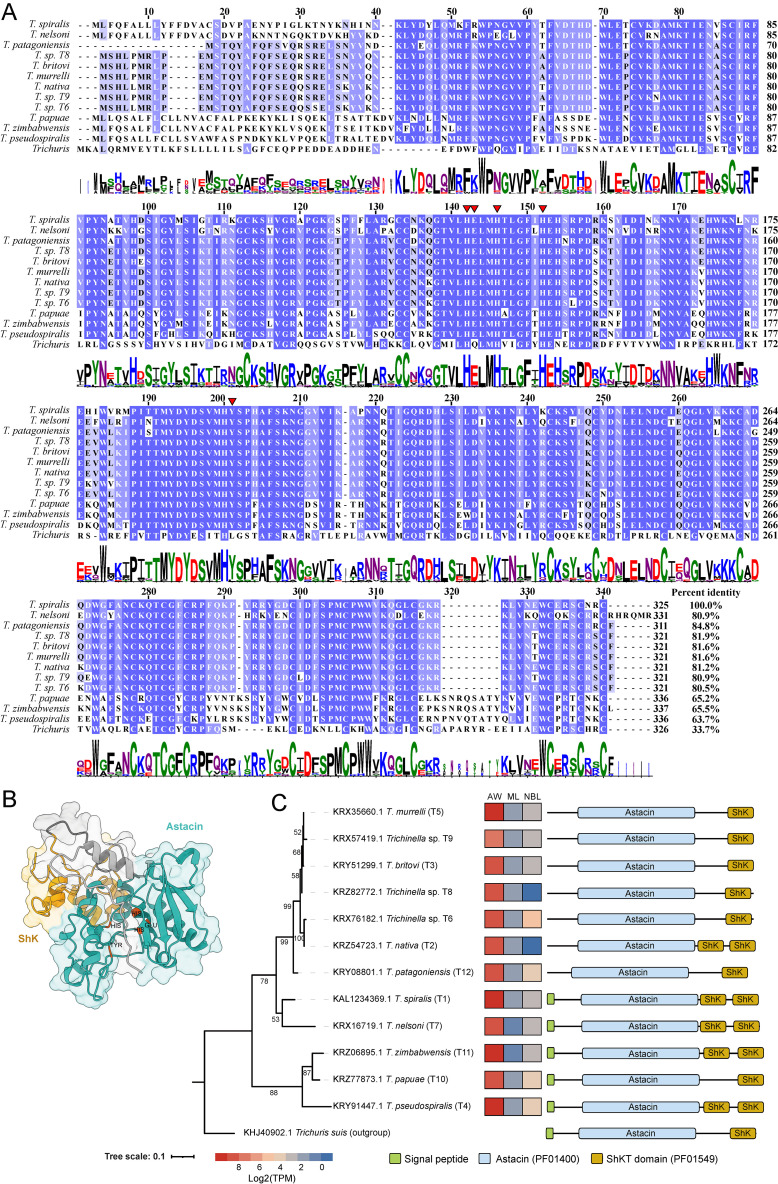
Bioinformatic characterization of the TsNas14 protein. A. Identical and similar residues are marked with blue gradient shading. Putative active site residues (137H, 138E, 141H, 147H, and 196Y) are marked with red triangles. The percentage at the end of each sequence indicates the similarity of the sequence to TsNas14. B. The cyan region in the 3D structure represents the Astacin domain, the yellow part represents the ShK domain, and the gray part represents the domain-free region. C. The phylogenetic tree of TsNas14 was inferred using the maximum likelihood. The heat map blocks of each branch indicate the relative mRNA expression of the Nas-14 gene in the three growth stages (AW, ML, and NBL) of *Trichinella*. A schematic representation of the domain linear position at the end of the branch demonstrates domain differences between the Nas-14 of different species.

The total CDS length of TsNas14 is 978 bp, encoding 325 amino acids, with a molecular weight of 37.86 kDa and an isoelectric point of 8.61. The N-terminal region has a signal peptide (AA 1–17), an astacin domain (AA 52–235, PF01400), and two ShK domains (AA 291–325, PF01549) and the Astacin domain contains five active residues (137H, 138E, 141H, 147H, and 196Y). The TsNas14 has > 80% homology with encapsulated species/genotypes, ~ 65% homology with nonencapsulated species (Fig 1A), and 33.5% homology with the outgroup species (*Trichuris suis*). These results indicate that TsNas14 is highly conserved in the *Trichinella* genus.

The AlphaFold 3 results of the three-dimensional structure of TsNas14 (no signaling peptide) returned a pTM score of 0.71, proving that the model structure was reliable. In the TsNas14 model, the ZnMc astacin-like domain consists of multiple α-helix motifs and β-folded sheets, and the active residue site locates in a pock-like structure (Fig 1B). The quality of the 3D structure was also evaluated using the SAVES v6.1 server and 78.25% of TsNas14 residues had an averaged 3D-1D score >= 0.1 while the overall quality factors of TsNas14 were 95.139.

Phylogenetic analyses of homologous proteins showed that TsNas14 is conserved among *Trichinella* species and is most closely related to *T. nelsoni* (Fig 1C). Published transcriptome data was used to investigate the transcriptional levels of TsNas14 in the different developmental stages of *T. spiralis*, revealing that the expression level of TsNas14 was highest in the adult or the enteric infection stage (Fig 1C). This protein expression pattern was also present in the life histories of other sister species (Fig 1C).

### 3.2. rTsNas14 had metalloproteinase activity

**Fig 2:**
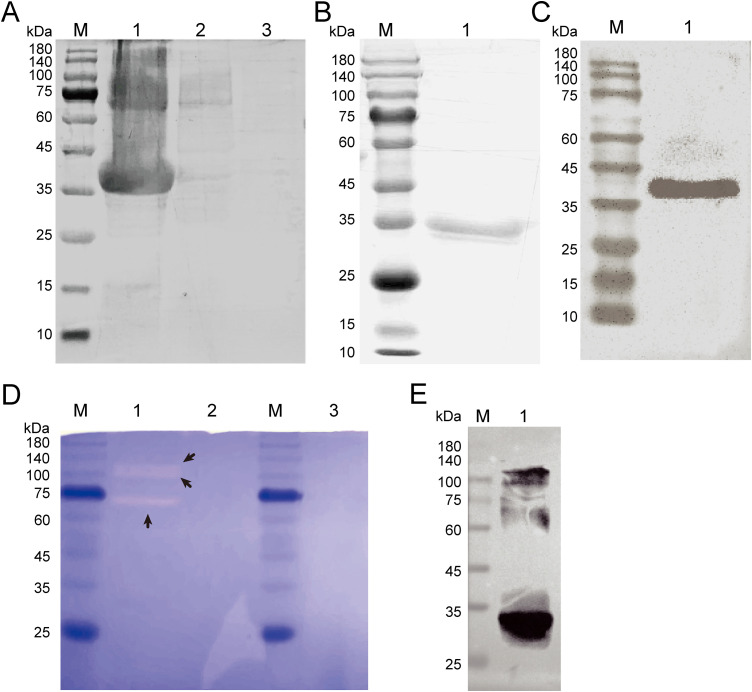
Expression and purification of rTsNas14 and enzyme activity assay. A. SDS-PAGE results for rTsNas14. Lane M: Protein marker. Lane 1: recombinant *E. coli* BL21 (DE3) pET-28a/TsNas14 lysate precipitation after 1 mM IPTG induction at 37°C; Lane 2: pET-28a/TsNas14 lysate before induction; Lane 3: pET-28a/TsNas14 lysate supernatant after induction. B. Purified rTsNas14 with Ni-NTA. C. Western blot analysis showed that rTsNas14 could be recognized by anti-His-tag McAb. D. The activity of rTsNas14 was detected by gelatin zymography. Lane M: Protein marker. Lane 1: Renatured rTsNas14. Transparent bands at ~70, ~ 100 and ~120 kDa can be observed after the degradation of gelatin; Lane 2: Supernatant of *E. coli* with the empty vector after IPTG induction; Lane 3: rTsNas14 with 1,10-phenanthroline. E. Western blot analysis based on non-denaturing electrophoresis showed that His-Tag antibodies could recognize specific bands.

The rTsNas14 (no signaling peptide) was expressed using a pET-28a vector, and the SDS-PAGE results showed that most of the proteins were expressed as inclusion bodies (Fig 2A). As expected, the molecular weight of the purified rTsNas14 protein was approximately 38.7 kDa (Fig 2B). Western blot results also showed that rTsNas14 could be recognized by His-Tag antibodies (Fig 2C).

The gelatin zymography results indicated that transparent bands appeared at ~70, ~ 100, and ~120 kDa compared with the control group (Fig 2D) and that 1,10-Phenanthroline (a metal ion chelating agent) could effectively inhibit the activity of rTsNas14. The results of the non-denaturation electrophoresis showed that all three bands were recognized as rTsNas14 by His-Tag antibodies (Fig 2E), suggesting that rTsNas14 was active in the form of an oligomer.

### 3.3. Temporal and spatial expression characteristics of TsNas14 protein

**Fig 3:**
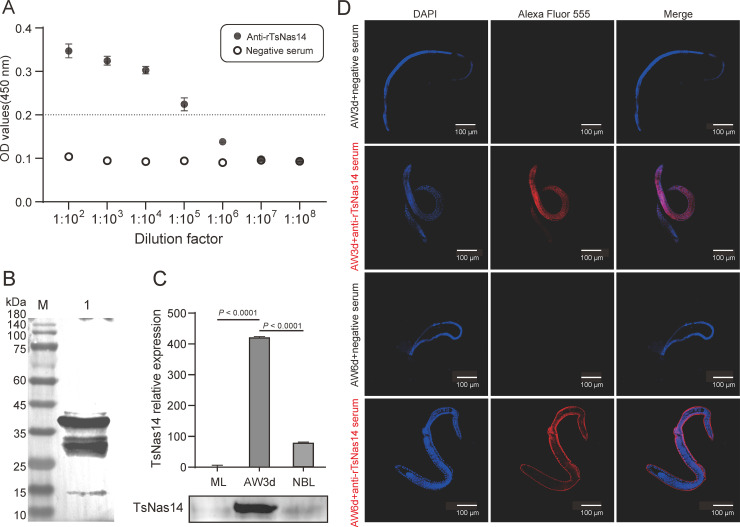
Spatiotemporal expression characteristics of TsNas14. A. Titer of rabbit Anti-rTsNas14 antibody. B. Western blot analysis showed that rTsNas14 could be recognized by Anti-rTsNas14 serum. C. qPCR and Western blot analyses showing the expression levels of rTsNas14 mRNA and protein during the different life stages of *T. spiralis*. The concentrations of all protein samples were quantified before analysis, D. Fluorescence localization of TsNas14 at different stages: nuclei stained with DAPI in blue and TsNas14 in red. ST: stichosome; OV: ovary; CU: cuticle; HY: hypodermis.

The serum anti-rTsNas14 IgG titer seven days after the last vaccination was 1:10^5^ (Fig 3A) and western blot analysis revealed a distinct band at the expected molecular weight (~38 kDa), demonstrating that the anti-rTsNas14 serum specifically recognized the purified rTsNas14 protein (Fig 3B). Additionally, several non-specific bands below 35 kDa were observed, potentially indicating protein degradation and suggesting that rTsNas14 has inherent instability.

The expression levels of TsNas14 in the ESPs of *T. spiralis* were assessed using anti-rTsNas14 serum and a significantly increased expression of TsNas14 protein was observed in the ESPs of the AW3d stage (Day 3 after infection) compared to the ML and NBL stages, which is consistent with the qPCR analysis results (Fig 3C).

The immunofluorescence detection results also showed that TsNas14 was expressed in multiple regions of the AW3d parasite, including the stichosome, ovary, cuticle, and hypodermis. However, during the AW6d stage (Day 6 after infection), its expression was restricted to the cuticle (Fig 3D).

### 3.4. rTsNas14 are involved in invading the intestinal barrier by degrading tight junction proteins

Members of the zinc metalloprotease family degrade collagen and the extracellular matrix and reports have shown that these proteins can promote the colonization and invasion of intestinal helminths [16]. Considering that the AW needs to break through the intestinal epithelial barrier to migrate and mate, it was speculated that the TsNas14 protein may interact with the intestinal epithelial barrier to assist *T. spiralis* invasion.

**Fig 4:**
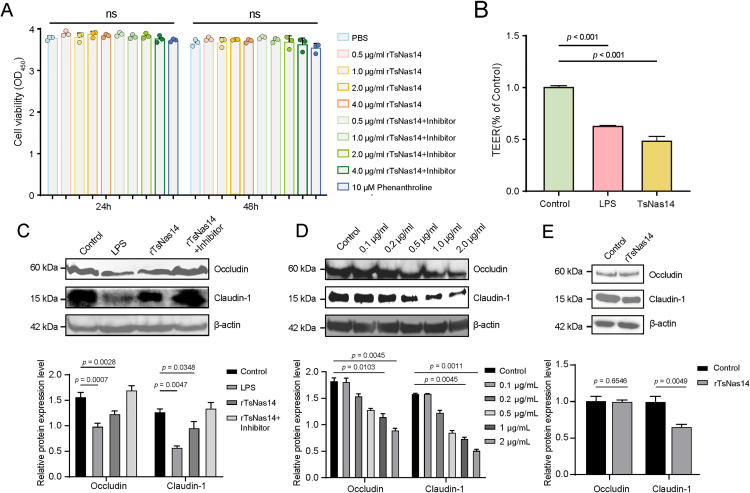
Effect of rTsNas14 on the intestinal barrier model. A. Cytotoxicity of different concentrations (0.5 μg/mL, 1.0 μg/mL, 2.0 μg/mL, and 4.0 μg/mL) of rTsNas14 on Caco-2 cells; B. Effect of rTsNas14 on TEER in the intestinal barrier model, Control was negative control and LPS was positive control; C. For the inhibitory effect of rTsNas14 on Occludin and Claudin-1 in Caco-2 cells, β-actin from *T. spiralis* was used as an internal reference protein. D. The inhibitory effect of rTsNas14 became stronger with the increase of rTsNas14 concentration. E. Western blot analysis demonstrated that rTsNas14 directly influences the levels of Claudin-1 protein.

To verify this hypothesis, active rTsNas14 was incubated with Caco-2 cells and the CCK-8 assays demonstrated that rTsNas14 at concentrations <4 µg/mL had no cytotoxic effects on the Caco-2 cells (Fig 4A). However, intestinal barrier integrity assessment experiments using 1 µg/mL rTsNas14 revealed that both rTsNas14 and LPS significantly reduced TEER values (Fig 4B). Western blot analysis also showed that co-incubation with rTsNas14 suppressed the expression of occludin and claudin-1 in the Caco-2 cells (Fig 4C), with this inhibitory effect being concentration-dependent (Fig 4D). Importantly, after the addition of inhibitors, rTsNas14 lost its inhibitory effect on the expression of the occludin and claudin-1 proteins. Notably, while rTsNas14 incubation did not directly degrade occludin, it demonstrated significant proteolytic activity against claudin-1 (Fig 4E). These findings collectively support the hypothesis that TsNas14 may facilitate parasite invasion through the degradation of tight junction proteins.

### 3.5. TsNas14 siRNA treatment decreased the infection ability of *T. spiralis*

**Fig 5:**
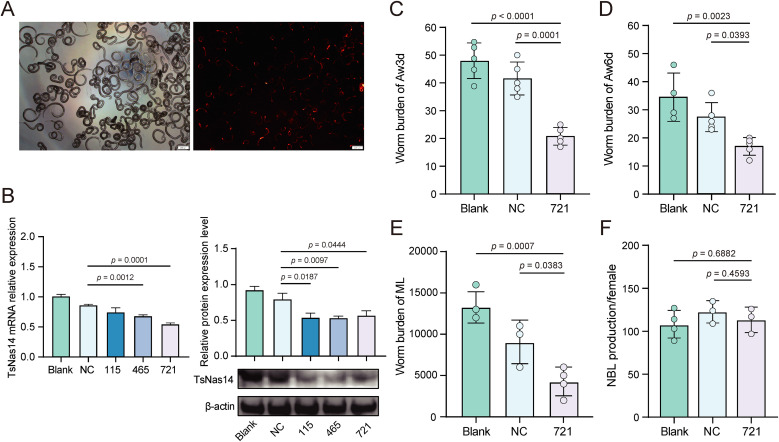
Effect of RNAi inhibition of TsNas14 expression on *T. spiralis* infection. A. Control siRNA with cy3 red fluorescence was transfected into ML. B. Expression of TsNas14 at the mRNA and protein levels after siRNA transfection in ML (115: TsNas14-sirNA-115; 465: TsNas14-siRNA-465; 721: TsNas14-sirNA-721). TsNas14-siRNA-721 reduced the worm burden of AW3d (C), AW6d (D), and ML (E) in mice. F. TsNas14-siRNA-721 did not significantly affect the ability of females to produce NBL. “ns” indicates no statistically significant difference compared with the control.

To investigate the effects of TsNas14 on invasion and survival of *T. spiralis*, RNAi technology was used to silence TsNas14 gene (Fig 5A), and compared with the blank control, TsNas14 transcription levels in the TsNas14-siRNA-721 and TsNas14-siRNA-465 groups were significantly inhibited by 36.95% and 21.15%, respectively (Fig 5B). Similarly, there were consistent results at the protein expression level. Based on these results, TsNas14-siRNA-721 was selected for subsequent experiments.

We artificially infected mice using ML in which TsNas14 expression was silenced and subsequently measured the infestation levels of AW and ML at 3, 6, and 35 dpi. The results indicated that, compared with the blank control, the number of AW3d, AW6d, and ML worm burden in the TsNas14 silenced group was significantly reduced (Fig 5C–5E). This suggests that silencing TsNas14 notably reduces the parasite’s survival and development in both adult and larval stages.

Additionally, we evaluated the fecundity of AW6d after TsNas14 gene silencing and the data demonstrated that the fecundity of AW6d post-silence was not significantly different from the blank control group (Fig 5F). These findings collectively validate that TsNas14 plays a crucial role in parasite invasion and colonization, but does not significantly affect the parasites reproduction.

## 4. Discussion

*T. spiralis*, an ancient parasite categorized within the nematode clade I [34], undergoes a critical niche transition during its life cycle as it migrates from the gut to blood or lymphatic vessels to invade the skeletal muscle of the whole body [4]. During this process, *T. spiralis* needs to break through the intestinal barrier, the basal layer, the walls of blood vessels or lymphatic ducts, and the muscle membrane several times. As with *Ascaris lumbricoides* and tapeworms, this process often causes severe symptoms due to larval migration and subsequent muscle invasion, such as myalgia, fever, and edema [35]. However, some in vitro studies have shown that *T. spiralis* is not as motile as free-living nematodes [4] despite being similar in size, and that adult *T. spiralis* can even have difficulty breaking through soft solid agar media. Some parasites, such as *Schistosoma japonicum* and *Onchocerca volvulus*, invade their host with intense mechanical movements that destroy large amounts of tissue and cause strong immune response [36,37]. However, over the course of a long period of adaptive evolution, the smaller *T. spiralis* seems to have chosen softer invasion methods to avoid an immune overreaction, which may have favored its long-term parasitism. In support of this, previous studies have shown that the ESPs of *T. spiralis* contains a variety of proteases that can directly or indirectly disrupt biological barriers to assist invasion [6,38–41].

The Nas protein family is one of the representative families in *T. spiralis* secretions and is expressed throughout its life cycle [8]. In this study, we characterized one of the zinc metalloproteinases Nas-14, namely TsNas14, which is highly expressed in the AW stage of *T. spiralis*. Multiple sequence alignments indicate that TsNas14 is highly conserved within the genus, but has low homology with neighboring genera, suggesting that it may be an important functional protein that differentiated early from the common ancestor of *Trichinella*. The 3D structure showed that the astacin domain of TsNas14 formed a pocket-like spatial structure with the active site located in the center of the pocket. The oligomer form of rTsNas14 demonstrated enzymatic activity capable of cleaving gelatin, likely due to an increased number of astacin active sites resulting from the oligomerization of TsNas14.

Within the *C. elegans* astacin protein family, proteins characterized by Astacin/ShK domains are classified under the Nas class II subgroup that encompasses a total of 10 genes, extending from Nas-6 to Nas-15 [42]. The TsNas14 protein features two ShK domains at its C-terminus, demonstrating structural homology with the *C. elegans* Nas-14 gene. Although the Nas-14 gene has been reported in various parasitic nematodes and is considered a promising biomarker [43], functional studies on this gene remain limited. Combinations of Astacin and ShK domains are common in venomous organisms and parasitic nematodes [44] and these domains play critical roles in processes such as predation, defense, and host-parasite interactions. The astacin domain typically exhibits metalloproteinase activity, aiding in the breakdown of extracellular matrix components, which can facilitate invasion or digestion. On the other hand, the ShK domain is known for its ability to block potassium channels [45–47], affecting nerve transmission and immune responses, which can be crucial for evading host defenses or subduing prey. This functional domain synergy makes their combination particularly effective for the ecological and survival strategies of venomous and parasitic species. Therefore, TsNas14 may also be involved in the invasion of host tissues and evasion of host immune systems by *T. spiralis*. This association warrants further investigation to elucidate the potential impact on host-pathogen interactions.

Since TsNas14 is a secreted protein that can be detected in the protein profile of ESPs, and thus come into direct contact with the host [8], combined with the temporal and spatial expression characteristics of this protease in worms, we hypothesized that TsNas14 is an important functional protein in the intestinal invasion process of adult *T. spiralis* worms. A study related to *T. spiralis* ESPs’ proteases found that metalloproteinase inhibitors can effectively reduce the degradation of tight junction proteins by ESPs [38], indicating that metalloproteinases are involved in the degradation process of tight junction proteins. Importantly, the disruptive effects of TsNas14 on tight junction protein and intestinal barrier models in vitro provide strong evidence for this hypothesis. Occludin is a membrane integrin that plays a role in the formation and regulation of the tight junction paracellular permeability barrier [48,49] and a decrease in occludin expression can lead to an increase in intercellular permeability. After TsNas14 treatment, the expression of occludin in Caco-2 cells decreased, but direct incubation of TsNas14 and occludin did not reduce the occludin content, indicating that TsNas14 may down-regulate occludin indirectly. Certain metalloproteinases, such as MMP-9 and MMP-2, have been demonstrated to degrade tight junction proteins [50]. The expression level of MMP-9 is regulated by multiple signaling pathways, including Notch1, NF-κB, and MAPK [51–53]. One potential mechanism is that TsNas14 may indirectly upregulate the expression of host matrix metalloproteinases (e.g., MMP-9) by activating these pathways (such as Notch1, NF-κB, and MAPK), thereby promoting occludin degradation. This potential effect may be attributable to TsNas14’s Astacin domain, as astacin-like proteins have been reported to activate NF-κB and MAPK pathways [54,55]. Claudin-1 is one of the main components of tight junction and is expressed in various tissues and organs [56]. The content of claudin-1 decreased significantly after the incubation of TsNas14 with the whole protein of the Caco-2 cells, indicating that TsNas14 could degrade claudin-1, and in a simulated intestinal barrier model in vitro, TsNas14 was able to lower TEER and destroy the barrier. Therefore, it is speculated that TsNas14 can destroy the intestinal barrier by down-regulating occludin and degrading the claudin-1 protein.

While our data clearly demonstrate the effect of rTsNas14 against claudin-1, the molecular mechanism underlying this regulatory remains to be fully elucidated. As a member of the zinc metalloprotease family, it is plausible that rTsNas14 directly cleaves claudin-1 at specific extracellular domain sites. Metalloproteases are known to target tight junction proteins like claudins [57,58]. Future studies employing techniques such as mass spectrometry analysis of claudin-1 degradation products or site-directed mutagenesis of potential cleavage sites could identify the exact cleavage sites and provide deeper mechanistic insights. Furthermore, the disruptive effect of TsNas14 on the intestinal barrier may also depend on other substrates or pathways. For instance, laminin, fibronectin and collagen, they are targets for metalloproteinases [59]. Proteomic and transcriptomic approaches could further elucidate the potential substrate specificity and regulatory mechanisms of TsNas14 in the intestinal barrier, which represents a promising research avenue.

Silencing the TsNas14 gene significantly reduced the worm burden in mice without affecting the fertility of female worms, suggesting that the TsNas14 gene plays a crucial role in parasitic invasion and not reproduction. However, a limitation exists in this study: the reduction in parasite burden resulting from TsNas14 gene knockdown may not solely depend on its direct impact on invasion. This is because analogous astacin zinc metalloproteinases have been reported to function in development, molting, and immune evasion [9,13,18]. Unfortunately, the lack of effective in vitro culture systems for observing *T. spiralis* molting and development restricts further exploration of TsNas14’s biological functions. Some indirect methods might be effective, such as performing heterologous expression in the *C. elegans* model [60,61], in order to investigate the functions of these difficult-to-culture parasitic proteins.

In conclusion, the zinc metalloproteinase nas-14 of a *T. spiralis* was characterized and its function was identified. The TsNas14 protein is highly expressed in the AW stages of *T. spiralis*, mainly localized in the stichosome, ovary, cuticle, and hypodermis. It exhibits metalloproteinase activity capable of degrading the claudin-1 protein and inhibiting the expression of the occludin protein in Caco-2 cells in vitro. Additionally, rTsNas14 can disrupt the intestinal barrier model while silencing the TsNas14 gene effectively reduces host infestation. These findings demonstrate that TsNas14 plays a critical role in mediating the intestinal invasion of *T. spiralis*, highlighting its potential as a promising candidate for vaccine development or therapeutic strategies.
